# Is Total Serum Nitrite and Nitrate (NOx) Level in Dengue Patients a Potential Prognostic Marker of Dengue Hemorrhagic Fever?

**DOI:** 10.1155/2018/5328681

**Published:** 2018-07-05

**Authors:** Maheshi Mapalagamage, Shiroma Handunnetti, Gayani Premawansa, Sharmila Thillainathan, Tharanga Fernando, Karunayokiny Kanapathippillai, Rajitha Wickremasinghe, Aruna Dharshan De Silva, Sunil Premawansa

**Affiliations:** ^1^Institute of Biochemistry Molecular Biology & Biotechnology, University of Colombo, No 90, Cumaratunga Munidasa Mawatha, Colombo 03, Sri Lanka; ^2^Colombo North Teaching Hospital, Hospital Inner Road, Ragama, Sri Lanka; ^3^Faculty of Medicine, University of Kelaniya, No. 06, Thalagolla Road, Ragama, Sri Lanka; ^4^Genetech Research Institute, No. 54, Kitulwatte Road, Colombo 08, Sri Lanka; ^5^Department of Zoology and Environment Sciences, Faculty of Science, University of Colombo, Cumaratunga Munidasa Mawatha, Colombo 03, Sri Lanka

## Abstract

Potential use of total nitrite plus nitrate (NOx) and nitrite (NO_2_^−^) separately as surrogate markers for serum nitric oxide in severe dengue and their longitudinal changes along with the progression of infection was studied. Deproteinized sera from confirmed dengue fever (DF, *n* = 145) and dengue hemorrhagic fever (DHF, *n* = 74) patients on admission—A, critical—C, discharge—D, and convalescence—CON stages and from age-gender matched healthy individuals (HC, *n* = 77) were taken to assess NO_2_^−^ and NOx levels using Griess and modified Griess assays. Serum NOx in DHFA was significantly lower compared to DFA (*p* < 0.001). HC had the lowest NOx and NO_2_^−^ compared to all patient categories (*p* < 0.001) except NO_2_^−^ in DF-CON and DHF-CON and NOx in DHF-CON. Serum NOx and NO_2_^−^ in DHF patients admitted on fever day 3 (DHFA-3) was significantly lower compared to DFA-3 (*p* < 0.05). Cut-off values of 4.46 *μ*M for NOx (91.3% sensitivity and 80.1% specificity) and 1.25 *μ*M for NO_2_^−^ (75.0% sensitivity and 73.3% specificity) were obtained for day 3 of fever. Serum NOx may be used as potential prognostic marker of DHF in patients presenting with DF in the early stage (on day 3 of fever) of the disease.

## 1. Introduction

Dengue fever is considered as one of the most important mosquito-borne viral infections in the world especially in tropical and subtropical countries [1]. The dengue virus belongs to the family Flaviviridae and genus *Flavivirus* and can cause dengue fever (DF) and more severe forms of the disease, dengue hemorrhagic fever (DHF) and dengue shock syndrome (DSS) [2].

Although the majority of dengue cases are not fatal, a minority of patients infected with dengue virus develops dengue hemorrhagic fever with high morbidity and mortality. The major pathophysiology of DHF is an acute increase in vascular permeability that leads to leakage of plasma through the endothelium [3, 4]. DHF in the critical stage is defined as the presence of one or more of signs of leakage including rising packed cell volume (>20%), pleural effusion, abdominal ascites, albuminaemia, etc. [5].

Dengue infection produces a broad spectrum of symptoms, many of which are nonspecific. Thus, to diagnose dengue viral infection, several laboratory methods have been implemented including virus isolation, detection of viral RNA (using reverse transcriptase polymerase chain reaction), detection of antigens (nonstructural protein 1 (NS1) of the virus), and serological diagnosis of anti-dengue IgM and IgG antibodies [3, 5]. Although there are well-established laboratory diagnostic methods to confirm the disease, most predictors of dengue disease severity are based on clinical information including pleural effusion, abdominal ascites, narrow pulse pressure, and rising packed cell volume (PCV, >20% from base level). However, the major problem is that all these severity signs are observed at the critical phase of the disease after the patient has progressed to severe disease. Therefore, the use of an early predictor or a prognostic indicator to identify dengue patients prior to progressing to the critical stage will be extremely useful to reduce disease morbidity and mortality.

Several studies have been carried out based on this concept of prognostic markers for severe dengue. Assessment of serum lactate dehydrogenase (LDH), creatine kinase (CK), and albumin levels at 48–96 hours after onset of fever has been reported to be positively correlated with severe dengue [6]. Another study revealed the mRNA expression of apoptosis and innate immune response-related genes were differentially regulated during the early stage of both mild and severe dengue patients [7]. However, the suitable biomarkers for early detection of severe dengue infection could be selected from the components of innate immune system since they are produced at an early stage of the disease compared to the acquired immune response.

Reactive oxygen species (ROS) and nitrogen species (RNS) are generated in the monocytes, macrophages, and many other immune cells during a viral infection, mainly to kill the viral load [8]. However, excessive production of these ROS and RNS makes the imbalance between these prooxidants and antioxidants, leading to oxidative stress that may cause many deleterious effects to the host [9]. Nitric oxide (NO), a highly reactive molecule, is considered to be a major prooxidant in the body which has the ability to diffuse through most cells and tissues [10]. In the presence of L-arginine, NADPH, and oxygen (O_2_), the enzyme nitric oxide synthase (NOS) produces NO, L-citrulline, and NADP [11]. NO is an important molecule to maintain normal physiological functions in the body such as vasodilatation, thrombosis, and neural activity. It also serves as a mediator for inflammation [12]. During an infectious disease, NO plays a pivotal role by fighting against the infectious agent nonspecifically [13]. NO has an inhibitory activity too on viral replication. NO can also suppress RNA synthesis in viruses and inhibits its activity resulting in a low viral load [14]. NO is a highly unstable molecule which quickly converts into more stable forms such as nitrite (NO_2_^−^) and nitrate (NO_3_^−^) collectively known as NOx. NOS enzyme has three isoforms, namely, the endothelial (eNOS), neuronal (nNOS), and inducible (iNOS) enzymes. eNOS and nNOS are constitutive, and iNOS is induced by cytokines and other proinflammatory stimuli. iNOS has been shown to generate higher concentration of NO than the other isoforms [15]. Previous studies have reported lower NOx levels in DHF compared to DF; the main reason for such depletion of NOx in DHF patients might be the removal of NOx due to plasma leakage [16–18]. In this study, we show longitudinal changes of NOx and NO_2_^−^ (on admission on different days of fever, at critical stage, on discharge, and during convalescence) with the progression of the disease.

## 2. Methodology

### 2.1. Recruitment of Patients

A total of 297 dengue-suspected patients were recruited for this study from wards 9 and 12 of the Colombo North Teaching Hospital (CNTH), Ragama, Sri Lanka, from June 2014 to May 2016. Almost all patients were residents of Gampaha district (1387 km^2^), Western province, Sri Lanka. Pregnant women, elderly patients (>65 years), and patients below 12 years were excluded from the study, and patients who gave a history of previous dengue or other Flavivirus infections, diabetes, cardiovascular diseases, asthma were also excluded.

### 2.2. Ethics Approval

Ethics approval for this study was obtained from the Ethics Review Committee, Faculty of Medicine, University of Colombo, Sri Lanka (EC-13-172). Informed written consent was obtained from each participant prior to sampling. For patients below 18 years, consent was obtained from the guardian who was older than 18 years.

### 2.3. Clinical Categorization

Clinical characterization of DF and DHF patients was done using the comprehensive 2011, World Health Organization guidelines for prevention and control of dengue and dengue hemorrhagic fever [5]. Patients with acute febrile fever with two or more of the following symptoms including headache, retroorbital pain, myalgia, arthralgia, skin rash, leucopenia (white blood cell count < 5000 cells/mm^3^), and mild thrombocytopenia (100,000 to 150,000 cells/mm^3^) were categorized as dengue fever.  Box 1 shows the clinical characterization of DHF patients.

All hematological and serum biochemical measurements of patients were recorded from admission to discharge. Day of fever in patients on admission was recorded considering the duration from the first day of fever.

### 2.4. Disease Confirmation and Blood Sample Collection

The initial blood sample was collected from dengue-suspected patients on admission. In order to confirm dengue, rapid immunochromatography test (NS1) (SD BIOLINE Dengue NS1 kit, Standard Diagnostics, Gyeonggi, South Korea) [19] and/or quantitative IgM ELISA was performed using a commercially available kit (Institut Virion\Serion GmbH, Warburg, Germany). Patients who were positive for either NS1 or IgM ELISA were considered as dengue patients. At this time, these patients were only diagnosed as dengue fever; some of these patients progressed to DHF.

After collecting a blood sample on admission, confirmed dengue patients were monitored and blood samples were collected from patients who showed confirmed evidence of plasma leakage approximately within 24 hours of the leaking phase; this was considered as the DHF sample at critical stage (DHFC). Patients who were admitted with signs of leaking were also included in DHFC category. Another blood sample was collected from dengue patients on the day of discharge. Convalescent samples were also collected on day 30 of fever from those who were available during follow-up. Consequently, all samples collected from patients were categorized as follows: DF on admission (DFA), DF on discharge (DFD), DF at convalescence (DF-CON), DHF on admission (DHFA), DHF at critical stage (DHFC), DHF at discharge (DHFD), and DHF at convalescence (DHF-CON).

### 2.5. Healthy Controls

Age, gender, and area-matched healthy individuals (*n* = 77) to DHF patients were recruited based on a negative history of previous dengue or any Flavivirus episodes and without fever during the week prior to sample collection. Since almost all patients were residents of Gampaha district (1387 km^2^), Western province, Sri Lanka, healthy individuals in the community were recruited from the Gampaha district with prior approval from the Regional Director of Health Services, Gampaha District, Sri Lanka. Individuals, who gave a history of previous dengue or other Flavivirus infections, diabetes, cardiovascular diseases, and asthma, were excluded from the study.

### 2.6. Serum Separation and Processing

Since serum nitrate is dependent on dietary factors [20], blood samples from all patients and healthy controls were collected in the morning between 0500 and 0600 h to minimize the dietary effect. Patients and healthy individuals were not kept fasting purposefully; however, they had taken their last meal ~8 h prior to the time of blood collection. Five milliliters of blood was collected by venepuncture into a sterile plain tube and serum was separated by centrifugation at 900*g* for 10 minutes, and clear sera were stored at −20°C.

Before measuring the serum NOx and NO_2_^−^ levels, all serum samples were deproteinized as previously described [21]. Ten microliters (10 *μ*l) of 1.5 g/ml ZnSO_4_ solution was added to 1 ml of serum. Mixture was thoroughly vortexed for 1 min and centrifuged at 10,000*g* for 15 minutes at room temperature (RT, 25°C). The supernatant was centrifuged again for 10 minutes, and clear deproteinized sera were immediately used for the assessment of NO_2_^−^ and NOx.

### 2.7. Measurement of Serum NO_2_^−^ and NOx Levels

Deproteinized sera (100 *μ*l) from each sample were added in duplicate into wells in a 96-well plate. This was followed by addition of 100 *μ*l of Griess reagent (0.1% N-ethylenediamine dihydrochloride and 1% sulphanilamide in 5% H_3_PO_4_) and incubated for 15 min at RT in dark. Optical density was measured at 540 nm (OD_540nm)_ using an ELISA plate reader (EL×800 Universal Microplate Reader, BioTek Instruments Inc., Canada).

Total concentration of serum NO_2_^−^ and NO_3_^−^, derivatives of NO, was measured using the modified Griess assay [21, 22]. Modification was made by adding 8 mg/ml VCl_3_ to serum in order to convert NO_3_^−^ into NO_2_^−^. An equal volume (70 *μ*l) of deproteinized sera, VCl_3_, and Griess reagent was added in duplicates into wells and incubated for 30 minutes at RT in dark and OD_540nm_ was measured. Twofold dilution series of NaNO_2_ (0.193–100 *μ*M) was used to plot the standard curve.

### 2.8. Data Analysis

The statistical analysis was performed using SPSS version 20.0. Kolmogorov-Smirnov (K-S) test was performed to determine the distribution of variables. Descriptive statistics are given as mean ± SD. Following statistical tests were performed where necessary: chi-squared test, Fisher exact test, paired *t*-test, independent sample Student *t*-test, one-way ANOVA with Bonferroni post hoc correction, and Pearson correlation. Receiver operating characteristic (ROC) analysis was used to obtain cut-off values for each test. Statistical significance was defined as *p* < 0.05.

## 3. Results

### 3.1. Clinical and Laboratory Data Analysis

**Figure 1:**
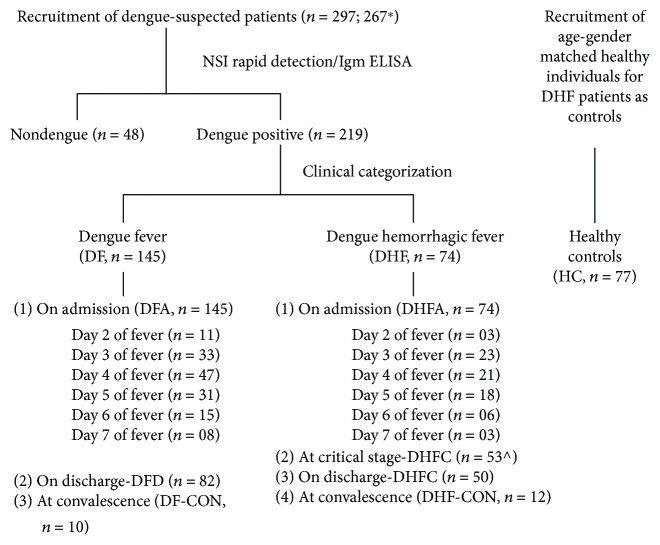
Sample collection plan. ^∗^Patients with comorbidities such as diabetes, asthma, and cardiovascular diseases and elderly patients (>65 years) (*n* = 30) were excluded from the study. ∧ includes 5 patients admitted having signs of plasma leakage and categorized as DHFC.

Of the 297 patients recruited, patients with comorbidities such as diabetes, cardiovascular disease, asthma, and elderly patients (>65 years) were excluded (*n* = 30). Among the 267 patients selected, 219 were laboratory confirmed as having dengue infection; out of which, 145 patients were clinically categorized as DF, and 74 patients were clinically characterized as DHF showing evidence of plasma leakage. Nondengue fever patients were excluded from the study (*n* = 48) (Figure 1). Most of the recruited patients were from the Gampaha district (97.9%) of the Western province of Sri Lanka. There were 5 patients who were admitted to the hospital having signs of plasma leakage and included in DHFC category (*n* = 5). No mortality was recorded in patients recruited for this study.

The highest percentage of DHF (31.1%) patients were admitted on day 3 of fever, and for DF patients, it was day 4 of fever (32.4%). The highest percentage of DHF (29.4%) and DF (34.4%) patients was discharged on days 9 and 8 of fever, respectively. Most DHF patients went into critical stage (DHFC) on the 7th day of fever (35.7%). Demographic data of all patients are depicted in Table 1.

Comparison of clinical, laboratory, and blood parameters of DF and DHF patients on admission is depicted in Table 2. Patients were monitored during the hospital stay, and records from admission to discharge of each patient were abstracted from the bed head ticket. Accordingly, in DHF patients, pulse pressure (mmHg), respiratory rate (breaths/min), and platelet count (×10^3^/mm^3^) were significantly low (*p* < 0.001) and leukocyte count (×1000), RBC (10^12^/L), and C-reactive protein (mg/dL) levels were significantly high compared with that of DF patients (*p* < 0.050). Comparison of symptoms in DF and DHF patients are depicted in Table 3. These symptoms were recorded on admission in each patient; patients were admitted between 2–6 days of fever. Significant number of DHF patients had recorded abdominal pain compared to DF patients (*p* = 0.013, Fisher exact test).

### 3.2. Changes of Serum NOx and NO_2_^−^ Levels in the Study Group

**Figure 2:**
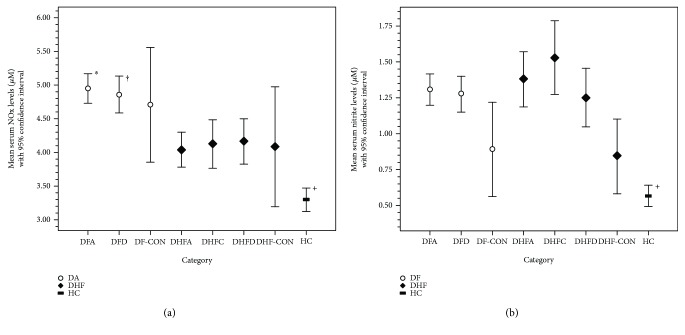
Levels of serum NOx (a) and NO_2_^−^ (b) in different patient and control groups. White circle: dengue fever (DF); black diamond: dengue hemorrhagic fever (DHF); black rectangle: healthy controls (HC). Figure 2(a) ^∗^Significant difference between DFA and DHFA, DHFC, DHFD, HC (*p* < 0.002). ^†^Significant difference between DFD and DHFA (*p* = 0.001), DHFC (*p* = 0.021), and DHFD (*p* = 0.037). ^+^Significant difference between HC and all patient categories (*p* < 0.020) except DHF-CON (*p* = 1.000). Figure 2(b) ^+^Significant difference between HC with all patient categories (*p* < 0.001) except DF-CON (*p* = 1.000) and DHF-CON (*p* = 1.000). One-way ANOVA with Bonferroni post hoc correction was used for comparing different study categories and paired sample *t*-test was done for repeated measures.

The lowest detectible value of serum NOx and NO_2_^−^ was determined as 0.193 *μ*M using the standard curve obtained from the twofold dilution series of NaNO_2_ (Y = 0.011X+0.000, *R* ^2^ = 0.999). The mean values of serum NOx and NO_2_^−^ in each category are given in Supplementary Table 1. According to the graph shown in Figure 2(a), NOx levels were significantly lower in DHFA (4.04 ± 1.12 *μ*M) compared to DFA (4.95 ± 1.32 *μ*M, *p* < 0.001) and DFD (4.86 ± 1.22 *μ*M, *p* = 0.001) but was significantly higher than HC (3.29 ± 0.68 *μ*M, *p* < 0.001). HC had the lowest NOx levels which was significantly lower than all patient categories (*p* < 0.050) except DHF-CON (4.08 ± 1.32 *μ*M, *p* = 1.000). Moreover, NOx levels in DHFD (4.16 ± 1.18 *μ*M) were significantly lower than in DFD (4.86 ± 1.22 *μ*M, *p* = 0.037).

There was no difference in NO_2_^−^ levels between DHFA (1.37 ± 0.87 *μ*M) and DFA (1.29 ± 0.64 *μ*M, *p* = 1.000). HC (0.56 ± 0.28 *μ*M) had the lowest serum NO_2_^−^ level which was significantly lower than all patient categories (*p* < 0.001) except DF-CON (0.88 ± 0.42 *μ*M, *p* = 1.000) and DHF-CON (0.84 ± 0.41 *μ*M, *p* = 1.000) (Figure 2(b)).

### 3.3. Changes of Serum NOx and NO_2_^−^ according to the Day of Fever

**Figure 3:**
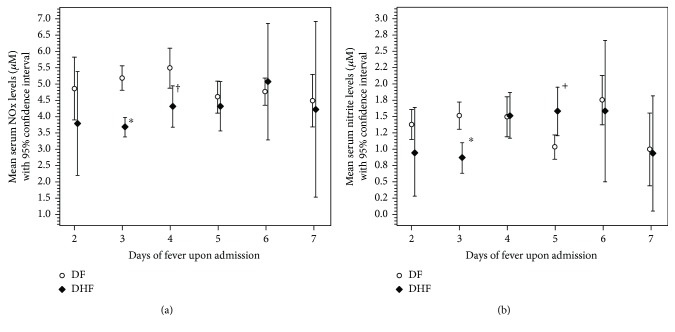
Changes of serum NOx (a) and NO_2_^−^ (b) levels in DHF and DF patients on admission with days of fever. White circle: dengue fever (DF); black diamond: dengue hemorrhagic fever (DHF); ^∗^ *p* < 0.001 between DHFA-3, DFA-3 and ^†^ *p* < 0.008 between DHFA-4 and DFA-4, and ^+^ *p* < 0.010 between DHFA-5 and DFA-5; independent Student *t*-test.

The prognostic value of serum NOx levels on admission of confirmed dengue patients (DFA and DHFA) was analyzed using receiver operating characteristic curve analysis (ROC). The ROC curve analysis of NOx levels resulted in an area under the curve of 71.7%; a cut-off value of 4.46 *μ*M gave a sensitivity of 70.8% and a specificity of 66.7%. However, considering the range of day of fever of patient cohort upon admission (2–6 days of fever), the NOx and NO_2_^−^ in DHFA and DFA were subgrouped according to the day of fever and compared (Figure 3).

**Figure 4:**
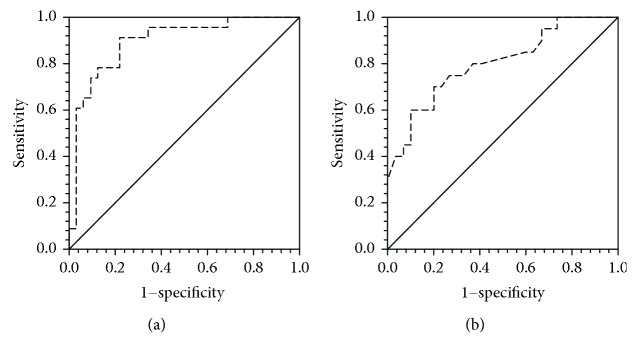
Receiver operating characteristic curves of serum NOx (a) and NO_2_^−^ (b) levels in confirmed dengue patients admitted on day 3 of fever.

Serum NOx levels in DHF patients admitted on day 3 of fever was significantly low compared to DFA on 3rd day of fever (DHFA-3, 3.67 ± 0.69 *μ*M; DFA-3, 5.16 ± 1.05 *μ*M; *p* < 0.001) (Figure 3(a)) and ROC curve analysis resulted 89.4% area under the curve and the cut-off value was determined as 4.46 *μ*M with 91.3% sensitivity and 80.1% specificity (Figure 4(a)).

Considering serum NO_2_^−^ levels of confirmed dengue patients on admission at day 3 of fever, the same trend was recorded as NOx (Figure 3(b)) where serum NO_2_^−^ in DHFA-3 (0.86 ± 0.50 *μ*M) was significantly lower than DFA-3 (1.48 ± 0.52 *μ*M, *p* < 0.001). The ROC curve analysis of NO_2_^−^ levels of confirmed dengue patients on admission at day 3 of fever resulted in an area under the curve of 80.2%; a cut-off value of 1.25 *μ*M gave a sensitivity of 75.0% and a specificity of 73.3% (Figure 4(b)).

DHF patients admitted on day 4 of fever (DHFA-4) had significantly lower NOx (4.29 ± 1.38 *μ*M) levels than DFA-4 (5.46 ± 2.07 *μ*M, *p* = 0.008). The ROC curve analysis resulted in an area under the curve of 71.0%; a cut-off value of 4.46 *μ*M gave a sensitivity of 65.2% and a specificity of 72.3%. Serum NO_2_^−^ levels in DHFA-4 (1.53 ± 0.80 *μ*M) and DFA-4 (1.49 ± 1.01 *μ*M) were similar (*p* = 0.926).

Serum NO_2_^−^ in DHFA-5 (1.57 ± 0.81 *μ*M) was significantly higher than DFA-5 (1.02 ± 0.52 *μ*M, *p* = 0.010).

According to Figure 3(a) and Figure 3(b), it is evident that serum NOx and NO_2_^−^ in DHFA is gradually increasing with days of fever, and in DFA, it shows a decreasing trend. Mean values of serum NOx and NO_2_^−^ in DFA and DHFA at different days of fever are depicted in Supplementary Table 1.

There were no association of serum NOx levels with age, BMI and clinical parameters (pulse rate, pulse pressure, respiratory rate, platelet count, hematocrit, leukocyte count, ALT, and AST) in both DF and DHF patients (Supplementary Table 2).

## 4. Discussion

Based on our study, it is evident that serum NOx levels in DHF patients were significantly low compared with that of DF patients regardless of the disease stage except for the convalescence stage. However, such decrement was not observed when comparing serum NO_2_^−^ levels in both categories. Both NOx and NO_2_^−^ levels of patients grouped according to their days of fever on admission had the same trend in DHF patients where it was significantly low at the early stages of fever (day 3 of fever on admission) and gradually increased with the days of fever when hospital admission was longer. Moreover, it was also observed that at early stage of the disease (day 3 of fever upon admission), the production of NOx and NO_2_^−^ is significantly low in DHF patients compared to DF patients. In addition, HC had the lowest levels of NOx and NO_2_^−^.

Our findings are comparable with previous findings where lower NOx levels were observed in DHF patients compared to DF patients, and healthy controls had the lowest levels [16, 17]; the possible reason for a lower level in DHF patients was attributed to plasma leakage. Our findings show that NOx levels are low in all three disease stages of DHF compared to DF. Serum NO_2_^−^ levels were low in DHF patients on admission at a very early stage of the disease (day 3 of fever) when plasma leakage is not evident. This shows that plasma leakage may not be the sole reason for the depletion of NOx. This type of categorization (patients on admission, critical stage, discharge, and convalescence) and testing of both NOx and NO_2_^−^ in these categories has not been done in previous studies. In this study, the dietary effect on serum NO, which can increase serum NO_3_^−^ levels, was minimized by collecting all samples fasting for 8 h.

Previous studies have indicated that NO can inhibit viral replication [14], and therefore, the antiviral effect of NO could protect the cells from damages caused by increased levels of cytokines induced by the viral infection. In DHF patients, the lower NO levels may have made a more favorable environment for the virus to flourish. This may have resulted in a high viral load which may have increased the adverse pathophysiologic process in the body leading to increased severity. Another study showed high viral load in DHF patients compared to DF patients [23]. Particularly, the authors of that study have shown high viral load in DHF patients in both acute and defervescence periods, suggesting that continued active viral replication or delay in clearance of viremia contributes to the pathogenesis of DHF. Therefore, our findings support this evidence showing the contribution of low NOx levels for the retention of the viremia throughout all disease stages. Another study has shown that the inhibitory effect of NO is related to the inhibition of DENV-2 RNA-dependent RNA polymerase (RdRp) domain of NS5 antigen with further suppression of viral RNA synthesis and viral structural proteins [24].

Previous studies have reported that NOx levels could be altered due to many other diseases including cardiovascular disease [25], asthma [26], diabetes [27, 28], Behçet's disease [29], systemic sclerosis [30], chronic hepatitis B virus [31], and skin allergy [32, 33]. NOx levels could also be changed due to aging [34] as well. Therefore, patients with cardiovascular diseases, asthma, and diabetes and elderly patients (>65 years) were excluded from this study. Further, there were no patients diagnosed for Behcet's disease, systemic sclerosis, chronic hepatitis B, and skin allergy in this study.

Findings of this study on the low levels of NOx and NO_2_^−^ in DHF patients in the early stage of the disease, that is, day 3 of fever, may be used as a probable prognostic marker to predict DHF. The major importance of these assays is the cost effectiveness and the availability results within a short duration. The simplicity of the assay makes it extremely feasible to be performed even in a rural hospital environment. However, the use of these tests as probable prognostic markers of DHF is limited to patients presenting at the early stages (day 3 or before) of illness. In this study, there were 31.9% (*n* = 70) of patients who presented early (day 3 or before). Dengue-suspected patients are encouraged to present to a medical practitioner during the early stage of the disease by health authorities. Further studies should be conducted with larger sample sizes to validate the findings of this study.

These findings suggest that the production of NO plays a protective role in early stage of the disease resulting in mild DF in some patients, while in others who are unable to produce significant amounts of NO, which is known to act against the virus at early stages, may result in low NOx and NO_2_^−^ levels leading to DHF. In addition, these findings show that the pathological effects of the innate immune system start at a very early stage of the infection and show the importance of early detection of the disease early and categorization of patients based on NOx and NO_2_^−^ levels. Differences in NO production in dengue patients may be due to the different genetic backgrounds.

## 5. Conclusion

Serum NOx and NO_2_^−^ levels during the acute phase of infection were significantly lower in DHF patients than DF patients in the early stages of the disease. DF patients may have been protected by the higher NOx and nitrite levels from progressing to DHF. Serum NOx levels in the early stages of the diseases (on day 3 of illness) may be used as a prognostic indicator for progression to DHF.

## Figures and Tables

**Box 1:**
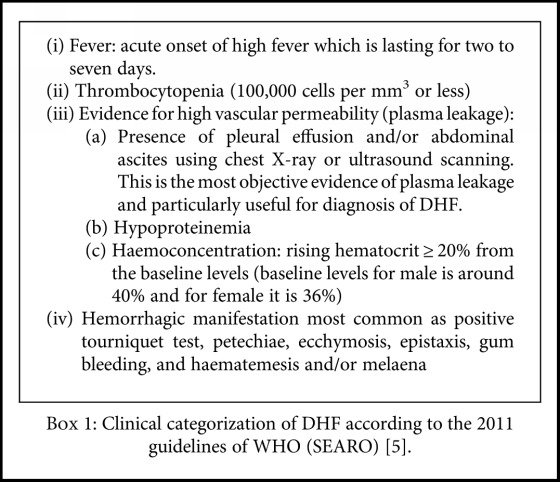
Clinical categorization of DHF according to the 2011 guidelines of WHO (SEARO) [5].

**Table 1:** Comparison of demographic data between DF, DHF patients, and HC.

Demographic data	Study group		
	DF (*n* = 145)	DHF (*n* = 74)	(HC *n* = 77)
Age (mean ± SD)	30.3 ± 14.3	29.2 ± 13.5	32.0 ± 11.8
Gender (male : female)	75 : 70	40 : 34	40 : 37
Body mass index (kg/m^2^)	21.45 ± 3.68	22.18 ± 5.70	23.63 ± 3.96

**Table 2:** Comparison of laboratory parameters between DF and DHF patients on admission.

Laboratory analysis of patient groups	DF		DHF		*p* value
	*N*	Mean ± SD	*N*	Mean ± SD	
Axillary temperature (°F)	145	99.9 ± 1.5	74	99.5 ± 1.1	0.075
Pulse rate (beats/min)	120	84.7 ± 7.1	68	85.8 ± 5.9	0.547
Pulse pressure (mmHg)	**114**	**37.8 ± 5.7**	**72**	**33.0 ± 4.1**	**<0.001**
Highest respiratory rate (breaths/min)	**124**	**21.5 ± 2.4**	**72**	**22.4 ± 1.8**	**<0.001**
Platelet count (10^3^/mm^3^)	**130**	**93.5 ± 38.1**	**68**	**61.9 ± 24.6**	**<0.001**
Hematocrit (%)	116	38.9 ± 5.1	73	39.2 ± 5.0	0.360
Leukocyte count (×1000)	**122**	**3.7 ± 1.6**	**73**	**4.5 ± 2.1**	**0.007**
Hemoglobin (g/dL)	78	13.7 ± 1.6	69	14.1 ± 1.8	0.180
Red blood cells (10^12^/L)	**75**	**4.7 ± 0.5**	**60**	**5.1 ± 0.6**	**0.018**
C-reactive protein (mg/dL)	**53**	**5.6 ± 4.2**	**40**	**10.2 ± 8.4**	**0.002**
Alanine aminotransferase (ALT) (U/L)	98	59.9 ± 51.6	70	85.96 ± 78.0	0.934
Aspartate aminotransferase (AST) (U/L)	97	94.3 ± 95.9	72	144.0 ± 147.5	0.284

Comparisons with significant differences are given in bold font.

**Table 3:** Comparison of clinical signs and symptoms of the disease in mild DF and severe DHF patients on admission.

Clinical signs and symptoms (%)	Percentage positivity (%)	
	DF (*n* = 145)	DHF (*n* = 74)
Headache	89.5	95.4
Retroorbital pain	58.3	69.3
Nausea/vomiting	72.7	75.5
Rash	6.6	13.1
Abdominal pain^∗^	**29.3**	**48.0**
Sore throat	18.2	28.5
Lethargy	82.5	89.4
Myalgia	84.6	91.8
Arthralgia	85	88.1
Red eyes	25.8	20.9
Ecchymosis/bruising	2.3	1.6
Bleeding	8.7	15.9

^∗^ *p* < 0.05 comparison between DF and DHF (Fisher exact test). Comparisons with significant differences are given in bold font.

## Data Availability

All relevant data are available within the manuscript and supplementary material files.

## Supplementary Materials

Supplementary Materials Supplementary Table 1: mean ± SD values of serum NOx and NO_2_^−^ in study group at different disease stages and at different days of fever upon admission. Supplementary Table 1 shows the mean ± SD values of serum NOx and NO_2_^−^ in the study group including 8 study categories (DFA, DFD, DF-CON, DHFA, DHFC, DHFD, DHF-CON, and HC) with respective sample numbers. Highest serum NOx was recorded in DF group compared to DHF and HC where HC has recorded lowest NOx and NO_2_^−^. Highest serum NO_2_^−^ levels were recorded in DHFC. Mean ± SD values of serum NOx and NO_2_^−^ in DF and DHF patients admitted on different days of fever ranging from day 2 to day 7 were also depicted in the Supplementary Table 1 with respective sample numbers, and there is a clear difference in the levels of serum NOx and NO_2_^−^ between DF and DHF patients particularly admitted at day 3 of fever. Supplementary Table 2: association between NOx levels and clinical parameters of DF and DHF patients (Pearson correlation). Supplementary Table 2 shows the Pearson correlation factor (*r* value) and the significance of serum NOx levels in DF and DHF patients with clinical parameters including pulse rate, pulse pressure, respiratory rate, platelet count, hematocrit, leukocyte count, alanine aminotransferase (ALT), and aspartate aminotransferase (AST) where no significant associations were found between each parameter tested.
