# Supplementary material for: Is Total Serum Nitrite and Nitrate (NOx) Level in Dengue Patients a Potential Prognostic Marker of Dengue Hemorrhagic Fever?
Source: Dis Markers. 2018 Jul 5;2018:5328681. doi: 10.1155/2018/5328681 (PMC6057279; doi:10.1155/2018/5328681)
Supplement: Supplementary Materials — Supplementary Table 1: mean ± SD values of serum NOx and NO2 − in study group at different disease stages and at different days of fever upon admission. Supplementary Table 1 shows the mean ± SD values of serum NOx and NO2 − in the study group including 8 study categories (DFA, DFD, DF-CON, DHFA, DHFC, DHFD, DHF-CON, and HC) with respective sample numbers. Highest serum NOx was recorded in DF group compared to DHF and HC where HC has recorded lowest NOx and NO2 −. Highest serum NO2 − levels were recorded in DHFC. Mean ± SD values of serum NOx and NO2 − in DF and DHF patients admitted on different days of fever ranging from day 2 to day 7 were also depicted in the Supplementary Table 1 with respective sample numbers, and there is a clear difference in the levels of serum NOx and NO2 − between DF and DHF patients particularly admitted at day 3 of fever. Supplementary Table 2: association between NOx levels and clinical parameters of DF and DHF patients (Pearson correlation). Supplementary Table 2 shows the Pearson correlation factor (r value) and the significance of serum NOx levels in DF and DHF patients with clinical parameters including pulse rate, pulse pressure, respiratory rate, platelet count, hematocrit, leukocyte count, alanine aminotransferase (ALT), and aspartate aminotransferase (AST) where no significant associations were found between each parameter tested. [file 5328681.f1.pdf]

Supplementary Materials

Supplementary Table 1: Mean±SD values of serum NOx and NO_2^- in the study group at different disease stages and at different days of fever upon admission.

Category	N	NOx (µM) Mean±SD	NO_2^- (µM) Mean±SD
DFA	145	4.95 ±1.32	1.29 ± 0.64
DFD	82	4.86 ± 1.22	1.26 ± 0.56
DF-CON	10	4.71 ± 1.11	0.88 ± 0.42
DHFA	74	4.04 ± 1.12	1.37 ± 0.87
DHFC	53	4.12 ± 1.23	1.65 ± 1.47
DHFD	50	4.16 ± 1.18	1.24 ± 0.71
DHF-CON	12	4.08 ± 1.32	0.84 ± 0.41
HC	77	3.29 ± 0.68	0.56 ± 0.28

Days of fever	Number of Patienets		NOx (µM)		NO_2^- (µM)	
	DF	DHF	DFA	DHFA	DFA	DHFA
Day 2	11	3	4.85 ± 1.42	3.78 ± 0.64	1.37 ± 0.32	0.95 ± 0.27
Day 3	33	23	5.16 ± 1.05	3.67 ± 0.69	1.48 ± 0.52	0.86 ± 0.50
Day 4	47	21	5.46 ± 2.07	4.29 ± 1.38	1.49 ± 1.01	1.53 ± 0.80
Day 5	31	18	4.59 ± 1.34	4.30 ± 1.47	1.02 ± 0.52	1.57 ± 0.81
Day 6	15	6	4.75 ± 0.73	5.08 ± 1.54	1.74 ± 0.62	1.57 ± 1.16
Day 7	8	3	4.47 ± 0.95	4.21± 1.08	0.99 ± 0.82	0.92 ± 0.55

Supplementary Table 2: Association between NOx levels and clinical parameters of DF and DHF patients (Pearson correlation)

Clinical parameters	DF		DHF	
	significance	r -value	significance	r- value
Pulse rate (beats/min)	0.323	0.233	0.552	0.155
Pulse pressure (mmHg)	0.428	0.182	0.178	0.180
Respiratory rate (breaths/min)	0.840	0.050	0.227	-0.279
Platelet count ($10^3/mm^3$)	0.627	-0.119	0.527	-0.160
Hematocrit (%)	0.354	0.189	0.185	-0.249
Leukocyte count (×1,000)	0.496	0.189	0.251	-0.216
Alanineaminotransferase (ALT)(U/L)	0.167	0.279	0.361	0.173
Aspartateaminotransferase (AST)(U/L)	0.586	0.133	0.738	0.070
